# Reduced CREB3L1 expression in triple negative and luminal a breast cancer cells contributes to enhanced cell migration, anchorage-independent growth and metastasis

**DOI:** 10.1371/journal.pone.0271090

**Published:** 2022-07-08

**Authors:** Paul Mellor, Stephanie Kendall, Shari Smith, Anurag Saxena, Deborah H. Anderson

**Affiliations:** 1 Cancer Research Group, University of Saskatchewan, Saskatoon, Saskatchewan, Canada; 2 Department of Pathology and Lab Medicine, Royal University Hospital, Saskatoon, Saskatchewan, Canada; 3 Department of Biochemistry, Microbiology and Immunology, University of Saskatchewan, Saskatoon, Saskatchewan, Canada; 4 Cancer Research, Saskatchewan Cancer Agency, Saskatoon, Saskatchewan, Canada; Turun Yliopisto, FINLAND

## Abstract

Women with metastatic breast cancer have a disheartening 5-year survival rate of only 28%. CREB3L1 (cAMP-responsive element binding protein 3 like 1) is a metastasis suppressor that functions as a transcription factor, and in an estrogen-dependent model of rat breast cancer, it repressed the expression of genes that promote breast cancer progression and metastasis. In this report, we set out to determine the expression level of CREB3L1 across different human breast cancer subtypes and determine whether CREB3L1 functions as a metastasis suppressor, particularly in triple negative breast cancers (TNBCs). CREB3L1 expression was generally increased in luminal A, luminal B and HER2 breast cancers, but significantly reduced in a high proportion (75%) of TNBCs. Two luminal A (HCC1428, T47D) and two basal TNBC (HCC1806, HCC70) CREB3L1-deficient breast cancer cell lines were characterized as compared to their corresponding HA-CREB3L1-expressing counterparts. HA-CREB3L1 expression significantly reduced both cell migration and anchorage-independent growth in soft agar but had no impact on cell proliferation rates as compared to the CREB3L1-deficient parental cell lines. Restoration of CREB3L1 expression in HCC1806 cells was also sufficient to reduce mammary fat pad tumor formation and lung metastases in mouse xenograft models of breast cancer as compared to the parental HCC1806 cells. These results strongly support a metastasis suppressor role for CREB3L1 in human luminal A and TNBCs. Further, the ability to identify the subset of luminal A (7%) and TNBCs (75%) that are CREB3L1-deficient provides opportunities to stratify patients that would benefit from additional treatments to treat their more metastatic disease.

## Introduction

Breast cancer is the most common cancer in women, and those with metastatic breast cancer have a disheartening 5-year survival rate of only 28% [1,2]. There are four major subtypes of breast cancer: luminal A (40–60%), luminal B (10–20%), HER2 (10–15%) and triple negative breast cancer (TNBC) (15–20%), which lack the three receptors that define the other subtypes [3]. HER2+ breast cancers overexpress the human epidermal growth factor receptor (HER2) and are treated with antibodies to HER2 such as trastuzumab [4]. Luminal breast cancers express receptors for estrogen (ER+) and/or progesterone (PR+). Luminal A (ER+, PR±, HER2-) breast cancers typically have a low proliferative capacity (low Ki67, a proliferative marker) and are often responsive to both endocrine and chemotherapy treatments [4]. Luminal B (ER+, PR±, HER2+) breast cancers have high Ki67 expression and usually respond to both endocrine and trastuzumab treatments, with variable responses to chemotherapy [4]. TNBC, most of which are basal-like, rely on chemotherapy as the primary treatment modality since no targeted therapies are currently available.

We previously characterized the role of CREB3L1 (cAMP responsive element binding protein 3 like 1) using derivatives of the estrogen-dependent rat mammary adenocarcinoma cell line R3230AC [5,6]. After selection of a highly metastatic subpopulation of cells through successive lymph node enrichment, we noted that the expression of CREB3L1 was significantly reduced [7] and went on to show that this loss contributed to their metastatic properties [6]. Using a syngeneic *in vivo* rat mammary tumor model, we also characterized tumor formation and metastasis. CREB3L1-deficient cells formed tumors at high frequencies, and most had lymph node metastases [6]. The same cells engineered to stably express CREB3L1 formed primary tumors at a reduced frequency at day 30, and by day 60 many of these regressed to a nearly undetectable size and none formed metastases (p<0.001) [6]. The results from this rat model of estrogen-dependent breast cancer [8] suggests that CREB3L1 can block tumor progression and play an important role in metastasis suppression.

CREB3L1 is a transcription factor, broadly expressed across many tissue types [9,10] and under low stress conditions resides in the endoplasmic reticulum as a transmembrane protein [11]. CREB3L1 protein levels are kept relatively low via HRD1-mediated constitutive ubiquitination and proteasomal degradation [12]. In response to cell stress, CREB3L1 transcription is increased and ubiquitination of the CREB3L1 protein is blocked resulting in increased CREB3L1 levels [12]. Subsequently, CREB3L1 is trafficked to the Golgi complex where it is cleaved by the site-specific proteases, S1P and S2P [11]. The soluble, mature and active CREB3L1 transcription factor translocates into the nucleus to regulate the expression of genes [11]. In rat breast cancer cells, chromatin immunoprecipitation and microarray analysis demonstrated that CREB3L1 negatively regulates genes that contribute to cell migration, invasion, angiogenesis and metastasis and positively regulates genes involved in tumor suppression [6].

In human breast tumor samples, about 30% have low levels of CREB3L1, primarily due to epigenetic silencing, which is also observed in breast cancer cell lines [13]. Reduced CREB3L1 expression is associated with higher tumor grade and more advanced metastatic disease [13]. Moreover, patients with reduced CREB3L1 expression have a shorter relapse-free survival as compared to patients with breast tumors expressing CREB3L1, particularly for the luminal A (p = 8.8 x 10^−6^) and TNBC (p = 0.038) subtypes [13]. In contrast, the survival of breast cancer patients with tumors that express amplified HER2 (HER2 and luminal B) do not appear to be significantly influenced by alterations in CREB3L1 expression [13]. Thus, the impact of CREB3L1 expression may be influenced by the genetic background (i.e. gene expression and/or mutational status) of the cancer cells. In this report, we set out to determine the contribution of CREB3L1-deficiency to the oncogenic cell properties in human breast cancer cells specifically of the luminal A and TNBC subtypes.

## Materials and methods

### CREB3L1 mRNA expression

The Gene Expression database of Normal and Tumor tissues 2 (GENT2) was used (April 9, 2021) to access gene expression information (http://gent2.appex.kr). GENT2 contains data from more than 68,000 samples compiled from public expression data sets that includes 72 different tissue types both normal and tumor, as well as tumor subtypes data. Search parameters used were: Subtype profile; Tissue: breast; Subtype: subtype; Gene symbol: CREB3L1. Normal breast tissue expression of CREB3L1 is from the GPL96 dataset. Dot plots were generated, and a one-way ANOVA followed by Tukey’s multiple comparison test was carried out using GraphPad PRISM software (version 9.1.0; GraphPad Software, Inc.), significance was defined as p < 0.05. TNBC and luminal A samples with CREB3L1 expression more than one standard deviation less than the median of the CREB3L1 expression in normal breast tissue were defined as having a Z score < -1 and were therefore defined as CREB3L1-deficient.

### Breast cancer cell lines and the generation of stable HA-CREB3L1-expression lines

Breast cancer cell lines used included basal TNBC (HCC1806, HCC70) and luminal A (HCC1428, T47D), obtained from the American Type Culture Collection (ATCC). Cells were authenticated by the supplier (http://www.ATCC.org) and cultured as recommended by ATCC for less than six months from the time of resuscitation. Clonal cell lines were generated that stably express triple hemagglutinin (HA)-tagged CREB3L1 (HA-CREB3L1). The plasmid encoding HA-CREB3L1 was generated by PCR amplification of the entire coding region of human CREB3L1 [6] and subcloned in-frame into an HA3 vector described previously [14]. The entire coding sequence was verified by DNA sequencing. Cells were transfected with the HA-CREB3L1 plasmid using lipofectamine 3000 (Thermo Fisher Scientific) according to the manufacturer’s directions and selected and maintained in geneticin (Thermo Fisher Scientific) to kill untransfected cells (100 μg/ml for HCC70, 400 μg/ml for HCC1806 and HCC1428, and 800 μg/ml for T47D cells). Single cell clones were expanded and tested for HA-CREB3L1 expression using an HA immunoblot analysis. Phase contrast photos (20x) were taken of each cell line using an EVOS 5000 Imaging System (Thermo Fisher Scientific) to show their phenotypic appearance. Vector control cell lines were generated by transfection of the HA3 vector and experiments were carried out after one week of geneticin selection.

### Immunoblot analyses

HA-CREB3L1 protein expression in the cell lines was visualized by immunoblot analysis as previously described [15]. Briefly, SDS-PAGE was performed using equivalent amounts of total protein (50 μg) as determined by Lowry (Sigma, TP0300). Samples were transferred to nitrocellulose and were probed with antibodies to HA (F-7 from Santa Cruz Biotechnology; mouse, 1 μg/ml) or β-actin (C-4 from Santa Cruz Biotechnology; mouse, 1:500), followed by infrared 800 nm dye-tagged secondary antibodies (LI-COR; 200 ng/ml). Blots were imaged with the LI-COR CLx Odyssey Infrared Imaging System (LI-COR). Blots shown are representative of the results seen from three independent experiments, using fresh cell lysates.

### Cell proliferation, migration and soft agar assays

Cell proliferation was measured by cell counting. Cells (2.5 x 10^5^) were seeded into three replicate 10 cm plates for each cell type and allowed to grow at 37°C and 5% CO_2_ for 24, 48 or 36 hours. At each time point, one plate of each cell type was washed in PBS, trypsinized, resuspended in 1 ml of cell culture medium. An aliquot of cells was counted using a Countess Automated Cell Counter (Thermo Fisher Scientific) and the total number of cells determined. Means ± SD from at least 3 independent experiments are shown.

For cell migration assays, cells were serum starved for 24 hours in media containing 0.5% fetal bovine serum (FBS) prior to migration. Cells were trypsinized, counted, and cells (2 x 10^5^) were placed in the top well of Boyden chamber (8 μm pore; Millipore Sigma) and allowed to migrate towards media containing 10% FBS for 4–24 hours as indicated. Negative controls were migrated towards 0.5% FBS containing medium as previously described [16]. Filters had the top unmigrated cells removed, then the migrated cells were fixed, stained and counted (nine high-powered fields) as previously described [16]. The migration index was determined by subtracting the cells migrated under control conditions (0.5% FBS) from the directional migration (10% FBS) values. Results are reported as means ±SEM from at least three independent experiment, each containing duplicate measurements.

Soft agar assays were used to assess anchorage-independent growth. Cells (5 x 10^4^) were seeded in soft agar and allowed to form colonies of cells for 21 days at 37°C and 5% CO_2_, as previously described [17]. Colonies were counted from nine high powered fields. Results are reported as means ±SEM from at least three independent experiment, each containing duplicate measurements. Representative images (20x) were taken using an EVOS 5000 Imaging System (Thermo Fisher Scientific).

### Mouse xenograft and tail vein metastasis assays

All animal procedures were carried out in accordance with the guidelines of the Canadian Council on Animal Care, and the regulations of the University of Saskatchewan Animal Care Committee (protocol number 20140030). Immune compromised female NOD/SCID/γ mice (8–10 weeks old) were anaesthetized with isoflurane and injected with HCC1806 or HCC1806+HA-CREB3L1 breast cancer cells (2 x 10^3^ in PBS), mixed 1:1 with Matrigel in the mammary fat pad (100 μl total volume). Each group contained 4–5 mice and 4 independent experiments were carried out for a total of 17–18 mice per group). Mice were monitored 3 times per week and primary tumor size (length and width) was measured using calipers. Tumor volumes were calculated as length x width^2^ x 0.5. Mice were euthanized for humane reasons when tumors reached the maximum of 1.5 cm in any dimension or due to observed signs of discomfort (weight loss, poor physical appearance, lack of mobility) or after 52 days. Primary tumors, lungs and livers were harvested, fixed in formalin (24–48 hours) and paraffin-embedded. Sections (5 μm) were stained with hematoxylin and eosin (H & E) [18]. No tumors were observed in the liver tissues. Lung metastases were evaluated by a pathologist and scored for the proportion (%) of the lung that was tumor bearing as compared to the lung size. Images were taken at 20x magnification using an Aperio virtual microscope (Leica).

Tail vein metastasis assays were carried out similarly. Immune compromised female NOD/SCID/γ mice (8–10 weeks old) were injected in the tail vein with a suspension of either HCC1806 or HCC1806+HA-CREB3L1 cells (2.5 x 10^5^ in PBS) [19]. Each group contained 4–5 mice and 2 independent experiments were carried out for a total of 8–10 mice per group. Mice were monitored 3 times per week and sacrificed after 46 days, determined as the optimal end point from pilot experiments. The lungs and livers were harvested, fixed, embedded and 5 μm sections were stained with H & E. No liver metastases were observed, however lung metastases were present in all animals and were evaluated, quantified and imaged as above.

### Statistical analyses

Data are expressed as the mean ± standard errors (SEM) from at least 3 independent experiments, unless otherwise indicated. The significance of changes was assessed using a student t-test (Microsoft Excel, version 16.47.1), with differences of p < 0.05 considered statistically significant.

## Results

### CREB3L1 expression is reduced in most TNBC tumor samples

**Fig 1:**
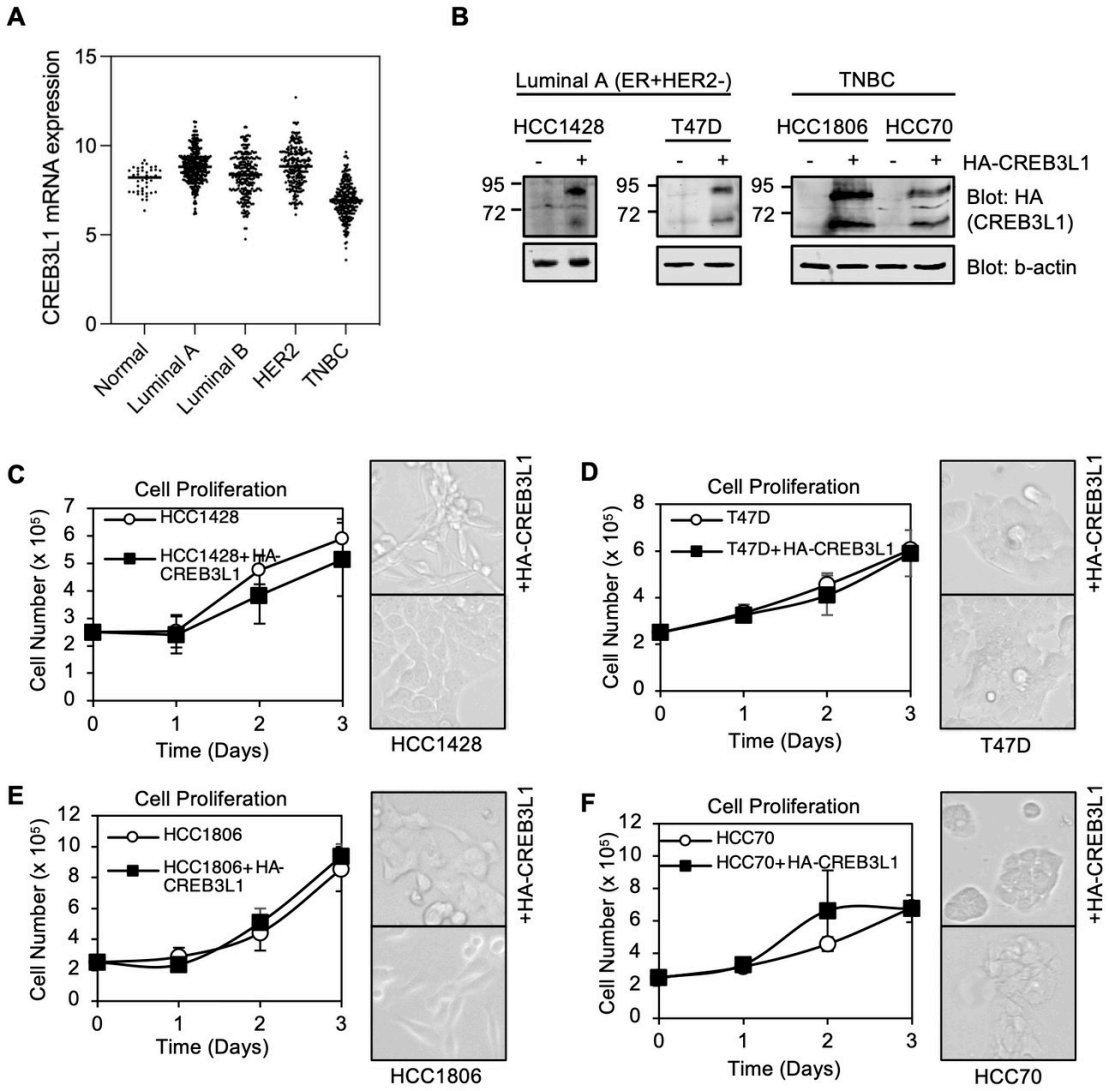
CREB3L1 expression is very low in TNBCs and the re-expression of HA-CREB3L1 alters cell phenotypes but not cell proliferation rates. (A) Dot blot of CREB3L1 mRNA expression in normal breast tissue as compared different subtypes of breast cancer (number of samples: 43, 379, 244, 230, 251). Data from the Gene Expression database of Normal and Tumor tissues 2—GENT2). Medians are indicated. (B) Equal amounts of cell lysates from the indicated human breast cancer cell lines that were stably expressing triple HA-tagged CREB3L1 (HA-CREB3L1) were resolved by SDS-PAGE, transferred to nitrocellulose and immunoblotted with antibodies to HA (CREB3L1) or β-actin (loading control). (C-F) Cell proliferation was determined by counting cells at the indicated time points after cells were seeded (2.5 x 10^5^ cells/ 10 cm plate). Mean ±SEM from at least 3 independent experiments, each with triplicate measurements. Phase contrast images of the cells (20x) are also shown.

To assess CREB3L1 expression in different breast cancer subtypes as compared to normal breast tissue, we accessed the Gene Expression database of Normal and Tumor tissues 2 (GENT2) [20]. This database provided CREB3L1 mRNA expression levels for a large number of breast tumor tissues classified into breast cancer subtypes: luminal A (ER+, PR±, HER2-), luminal B (ER+, PR±, HER2+), HER2 and TNBC (ER-, PR-, HER2-) as well as normal breast tissue (Fig 1A; S1 Table). Overall, luminal A (median 8.8) and HER2 (median 8.9) breast tumors showed a small but significant increase in CREB3L1 expression as compared to normal breast tissue (median 8.2) (p < 0.0001). The CREB3L1 expression in luminal B breast cancers (median 8.4) was similar to that in normal breast tissue. Most strikingly, TNBCs had the lowest CREB3L1 expression (median 6.9), significantly lower than all other subtypes of breast cancer as well as normal breast tissue (p < 0.0001). Although luminal A breast cancers generally showed an increase in CREB3L1 expression, nearly one-quarter of this subtype (24%; 92/379) had CREB3L1 expression levels less than the median expression seen in normal breast tissue, though only 7% (26/379) showed reductions more than one standard deviation below the mean for normal breast tissue (i.e. Z score < -1) (S1 Table). In contrast, the majority (90%; 226/251) of TNBCs had CREB3L1 expression levels below the median of normal breast tissue and most of these (75%; 187/251) showed significant reductions in CREB3L1 levels (Z score < -1) (S1 Table). These results suggest that CREB3L1-deficiency is a prevalent feature of TNBCs.

### Generation and characterization of CREB3L1-expressing luminal A and TNBC cell lines

We previously analyzed a large panel of human breast cancer cell lines and determined their endogenous CREB3L1 protein expression [13]. Most TNBC cell lines, like the TNBC tumor samples noted above, are CREB3L1-deficient, expressing little or no endogenous CREB3L1 protein [13]. Several luminal A breast cancer cell lines are similarly CREB3L1-deficient. Therefore, we selected four CREB3L1-deficient breast cancer cell lines for functional analysis, two basal TNBC cell lines (HCC1806 and HCC70) and two luminal A breast cancer cell lines (HCC1428 and T47D). To assess the impact of CREB3L1 loss versus expression on various cell properties, we generated stable clonal cell lines expressing triple hemagglutinin (HA-) tagged CREB3L1 (HA-CREB3L1) (Fig 1B). Both the full-length HA-CREB3L1 (~80 kDa) and the cleaved, active form of HA-CREB3L1 (~ 50 kDa) were observed.

The phenotypes of some of the CREB3L1-deficient parental breast cancer cell lines were altered by the expression of HA-CREB3L1, although the cell doubling times were not significantly impacted. This suggests that cell cycle progression and cell proliferation functions are not greatly impacted by CREB3L1 expression in breast cancer cells (Fig 1C–1F).

### Expression of CREB3L1 reduces cell migration and anchorage-independent growth of CREB3L1-deficient breast cancer cells

**Fig 2:**
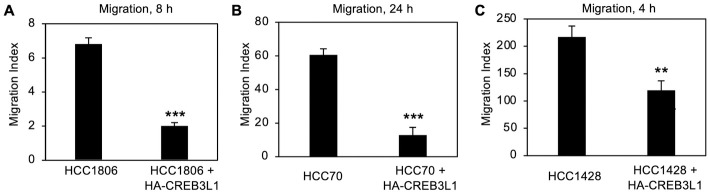
Expression of HA-CREB3L1 reduces cell migration in several CREB3L1-deficient human breast cancer cell lines. Cell lines ±HA-CREB3L1, HCC1806 (A) and HCC70 (B) and HCC1428 (C), were serum-starved (0.5% FBS) and then allowed to migrate for the indicated times through an 8-μm pore sized filter towards medium containing 10% FBS or 0.5% FBS. The number of cells migrated under both the conditions was quantified and the migration index was determined by subtracting the cells migrated under control conditions (0.5% FBS) from the directional migration (10% FBS) values. Mean ±SEM from duplicate samples in at least three independent experiments. **p < 0.01, ***p < 0.001.

To determine the impact of CREB3L1 expression on *in vitro* cancer cell properties, cell migration and growth in soft agar were assessed. Cell migration was analyzed for each cell line pair ±HA-CREB3L1, using Boyden chambers with an 8 μm pore size (Fig 2). No migration was observed for the T47D cells, likely since they are too large, 11–21 μm [21], so as not to fit easily through the pores. For the two TNBC cells, HCC1806 and HCC70, as well as the luminal A HCC1428 cells, expression of HA-CREB3L1 significantly reduced cell migration (Fig 2), in contrast to vector control cells that did not (S2A–S2C Fig). These results suggest that re-expression of CREB3L1 in CREB3L1-deficient human breast cancer cells blocks cell migration.

**Fig 3:**
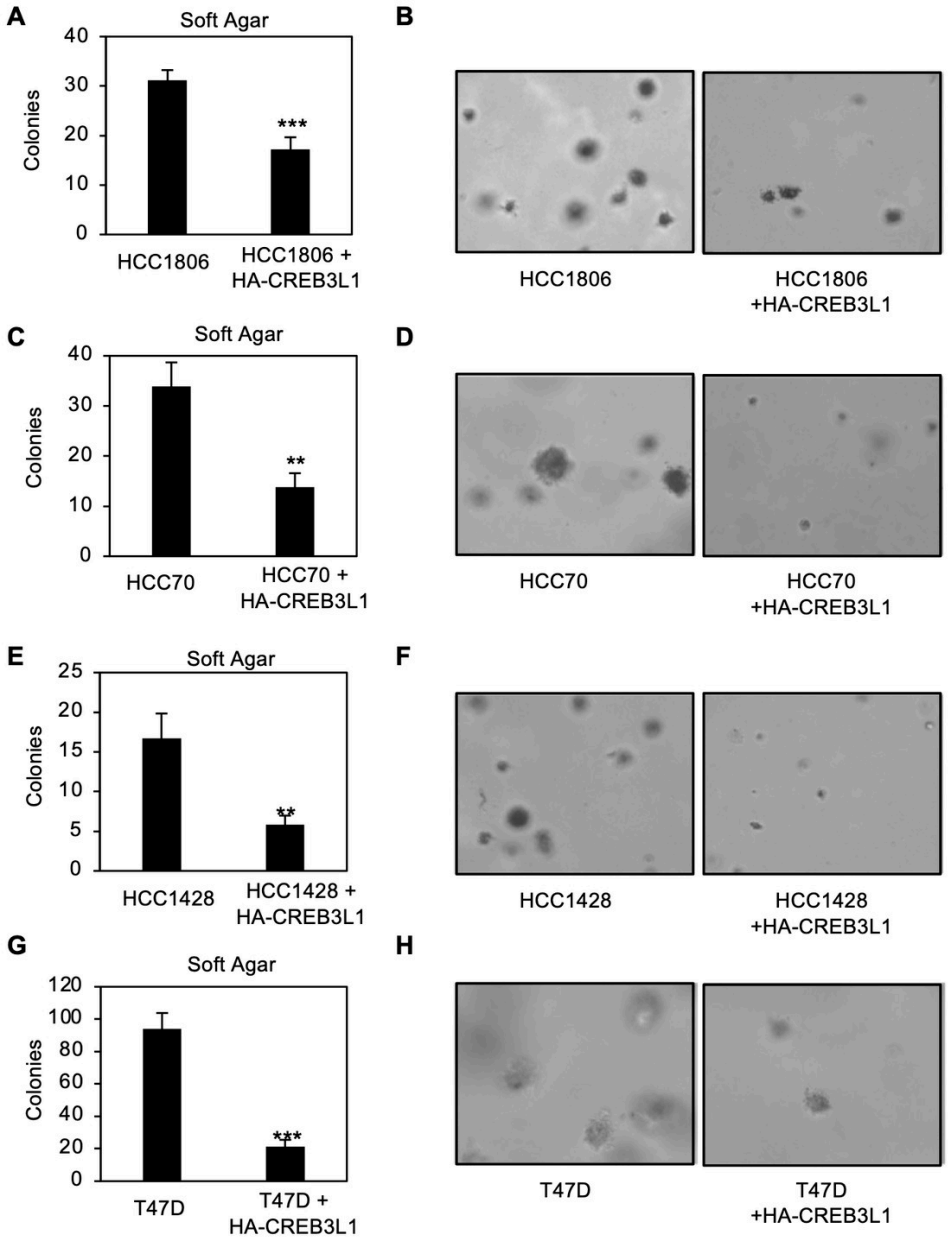
HA-CREB3L1 expression reduces anchorage-independent growth in soft agar across multiple breast cancer cell lines. Anchorage-independent growth was determined by growing the indicated cells (5 x 10^4^ cells seeded) in soft agar. The number of colonies formed after 21 days was counted (A, C, E, G). Representative images are shown (B, D, F, H). Mean ±SEM from at least 3 independent experiments. **p < 0.01, ***p < 0.001 as compared to the parental cells.

The impact of CREB3L1 expression on anchorage-independent growth was also evaluated (Fig 3). All four parental CREB3L1-deficient breast cancer cell lines formed numerous colonies in soft agar. The corresponding lines with HA-CREB3L1 expression showed a significant reduction in the number of colonies formed, unlike the vector control cells, suggesting that CREB3L1 expression inhibits anchorage-independent growth (Fig 3 and S2D–S2G Fig). These results suggest that CREB3L1-deficiency contributes to the cancer cell properties of cell migration and anchorage-independent growth.

### CREB3L1 expression reduces the growth of primary breast tumors and lung metastases in mouse xenograft experiments

**Fig 4:**
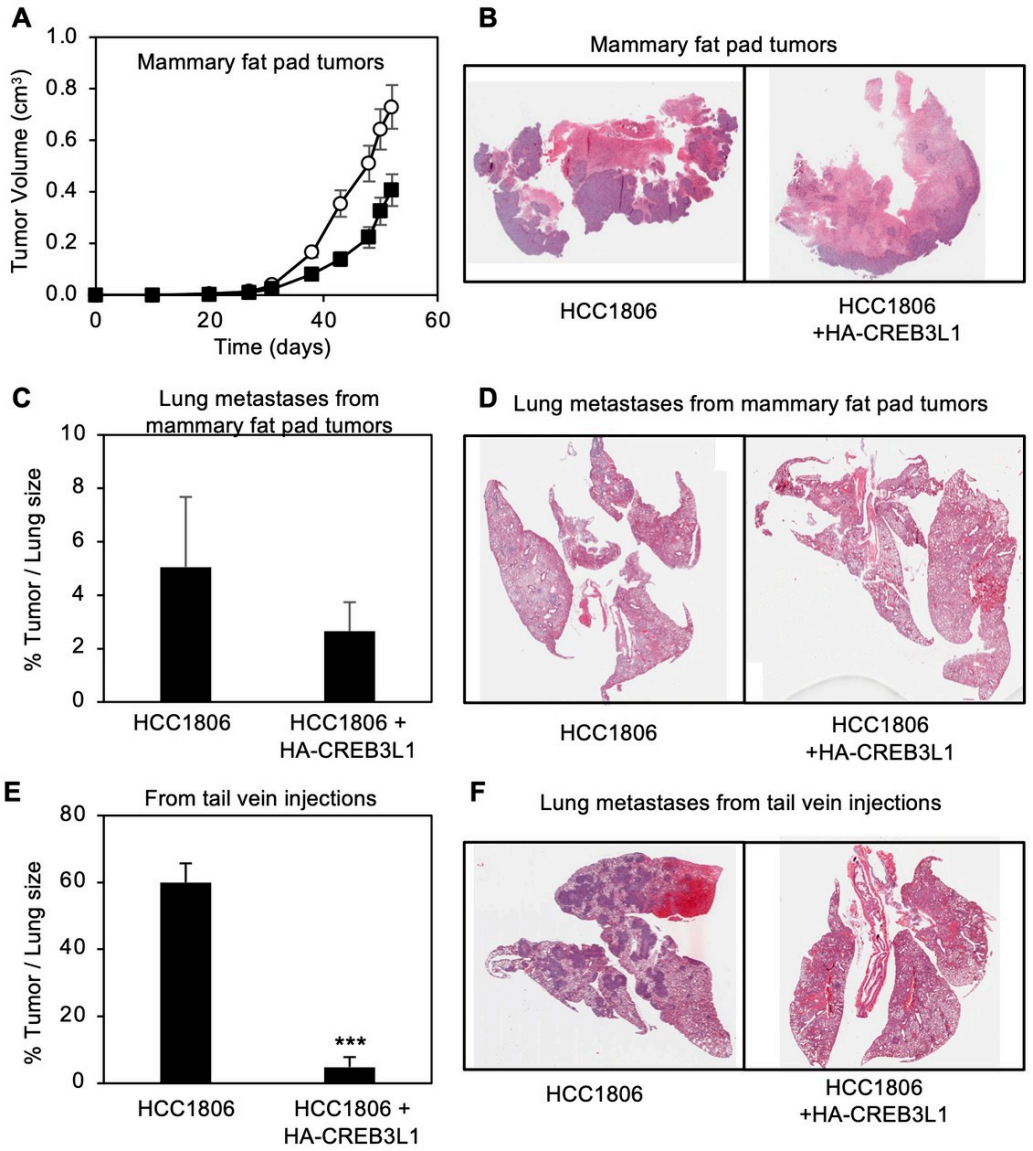
Restoration of CREB3L1 expression in CREB3L1-deficient HCC1806 cells reduces tumor growth and metastases. (A) HCC1806 cells (open circles; 2x10^3^) or HCC1806 +HA-CREB3L1 cells (filled squares; 2x10^3^) were injected subcutaneously into the mammary fat pads of female NOD/SCID/γ mice (17–18 mice total per group; 4–5 mice in each of 4 independent experiments) and tumor growth was measured over time. Mean ±SEM, p < 0.0001 between the 2 groups. (B) Tumors within the mammary fat pads were fixed in formalin, paraffin-embedded and sections were stained with H & E. (C-D) In two experiments, the lungs were excised from mice and fixed in formalin, paraffin-embedded and sections were stained with H & E (D). Samples were scored by a pathologist for the percentage of the lung that was taken up by the tumor (C). Mean ±SEM from 9–10 mice per group. p = 0.39 as compared to the parental cells. (E-F) HCC1806 ±HA-CREB3L1 cells (2.5 x 10^5^) were injected in the tail vein into female NOD/SCID/γ mice in a metastasis assay. After 46 days, the lungs were analyzed as in panels C and D. Mean ±SEM from 8–10 mice per group. ***p<0.001 as compared to the parental cells.

We next assessed the impact of HA-CREB3L1 expression on the tumorigenicity of HCC1806 cells. Female immune compromised (NOD/SCID/γ) mice [19] were injected in the mammary fat pad with either HCC1806 or HCC1806+HA-CREB3L1 cells and tumor growth was measured over time (Fig 4A). HCC1806 cells formed large primary breast tumors, whereas HCC1806+HA-CREB3L1 cells formed significantly smaller tumors (Fig 4A and 4B). We also examined the lungs and livers of these mice to identify and quantify metastases. No metastases were observed in the livers of these mice. Some lung metastases were found in mice with HCC1806 primary breast tumors and this number appeared somewhat reduced for the mice with HCC1806+HA-CREB3L1 primary breast tumors (Fig 4C and 4D).

To more directly measure the metastatic ability of these cells, tail vein injections of either HCC1806 or HCC1806+HA-CREB3L1 cells were also carried out in female NOD/SCID/γ mice. After 46 days, mice were euthanized, and lung and liver tissues were examined for metastases (Fig 4E and 4F). Again, no liver metastases were observed. The CREB3L1-deficient HCC1806 TNBC cells formed a large number of metastases in the lungs as compared to HCC1806+HA-CREB3L1 cells.

These results suggest that expression of CREB3L1 reduces the growth of primary breast tumors and blocks the formation of lung metastases, strongly supporting a role for CREB3L1 as a metastasis suppressor in human breast cancer cells.

## Discussion

Our previous work demonstrated that CREB3L1 can function as a metastasis suppressor in the estrogen-dependent rat mammary adenocarcinoma cell line R3230AC [6]. Using this model system, knockdown of CREB3L1 in CREB3L1-expressing breast cancer cells resulted in increased cell migration, invasion and anchorage-independent growth in soft agar [6]. Consistent with these results, re-expression of CREB3L1 in CREB3L1-deficient breast cancer cells reduced these cancer cell properties and blocked tumor growth and metastases in animals [6]. In this report, we provide additional evidence supporting a metastasis suppressor role for CREB3L1 in two human estrogen-dependent luminal A breast cancer cell lines (HCC1428 and T47D), as well as two TNBC cell lines (HCC1806 and HCC70). Expression of HA-CREB3L1 was sufficient to reduce both cell migration and colony formation in soft agar in CREB3L1-deficient human breast cancer cell lines. Tumor formation and metastases were also significantly reduced upon HA-CREB3L1 expression in TNBC HCC1806 cells. These results strongly support a tumor suppressor role for CREB3L1 in luminal A and TNBC.

Our findings are consistent with the survival data showing that both luminal A and TNBC breast cancer patients with CREB3L1-low tumors have a significantly worse prognosis, as compared to similar patients with CREB3L1-high tumors [13]. In contrast, breast cancers that express amplified levels HER2 (i.e. luminal B and HER2) do not appear to be impacted positively or negatively by CREB3L1 expression levels [13]. Thus, the impact of CREB3L1 expression can be influenced by the genetic background (i.e. gene expression and/or mutational status) of the cancer cells.

Similar subtype specific effects were described in a recent report which showed that CREB3L1 expression promoted metastasis in a very specific subtype of TNBC, the mesenchymal stem-like subtype, which only makes up about 5–10% of TNBCs [22]. This effect required the presence of three factors: endogenous CREB3L1 expression, activated PERK signaling and an epithelial-mesenchymal transition program [22]. Since ~75–90% of TNBCs are CREB3L1-deficient and the majority of TNBCs are of the basal subtype (~80%) [23], the effects observed in the mesenchymal stem-like subtype would not apply to most TNBCs. Thus, this appears to be the exception, and the more typical function of CREB3L1-deficiency in TNBCs, is to contribute to the metastatic phenotype as demonstrated here.

Overall, the majority of breast cancers show increased CREB3L1 expression as compared to normal breast tissue, however ~30% of breast cancers show decreased CREB3L1 expression as a result of epigenetic silencing [13]. A similar study by Rose *et al*. showed that CREB3L1 expression was also decreased in bladder cancer as a result of DNA methylation [24]. Re-expression of CREB3L1 in high-grade invasive bladder cancer cells reduced cell migration and the growth of colonies in soft agar, supporting a role for CREB3L1 as a tumor/metastasis suppressor in bladder cancer [24].

CREB3L1 is not typically mutated in cancers, but CREB3L1 functions as a metastasis suppressor in breast cancer [6,13] and bladder cancer [24]. CREB3L1 can be downregulated in these cancers by epigenetic silencing [13,24]. In prostate cancers, there are frequent structural rearrangements in the regulatory regions of the *CREB3L1* gene, that are predicted to contribute to tumorigenesis [25]. In these examples, CREB3L1 is functioning as a beneficial metastasis suppressor and its loss is associated with disease progression and poor prognosis.

## Conclusion

Thus, in this report we provide compelling evidence that strongly supports a role for CREB3L1 as a metastasis suppressor in luminal A and basal TNBC cells. Importantly, CREB3L1-deficiency is a frequent feature common to 75–90% of TNBC tumors, depending on what cut-off is used to define CREB3L1-deficiency. Further, we have identified a small subset of luminal A breast cancers (7–24%) that are CREB3L1-deficient, suggesting that this subgroup of luminal A breast cancers is likely to be more metastatic and could be identified and treated with chemotherapy agents to improve the outcomes of these patients. These results suggest that CREB3L1-deficiency could be a biomarker for metastatic cancers which could help identify patients at risk for treatment failure or early relapse, a group that may benefit from more aggressive treatment options or at least closer monitoring for disease recurrence.

## Supporting information

S1 Fig Raw images_Mellor & Kendall—Contains the full-size blots for Fig 1B. (TIF)

S2 Fig Cells transfected with vector control (HA) showed similar cell migration and growth in soft agar properties as untransfected parental cells. (A-C) The indicated cells were allowed to migrate towards 10% FBS through Boyden chambers for the indicated times and were fixed, stained and counted. Mean ±SEM from at least 2 independent experiments, each with duplicate determinations. (D-G) Growth in soft agar was determined after 21 days. Mean ±SEM from at least 2 independent experiments, each with duplicate determinations. In each case, there were no significant differences between the parental and vector control cells.
(TIF)

S1 Table CREB3L1 mRNA expression in normal breast and breast cancer subtypes. (XLSX)
