# Supplementary figures and images for: Reduced CREB3L1 expression in triple negative and luminal a breast cancer cells contributes to enhanced cell migration, anchorage-independent growth and metastasis
Source: PLoS One. 2022 Jul 8;17(7):e0271090. doi: 10.1371/journal.pone.0271090 (PMC9269740; doi:10.1371/journal.pone.0271090)

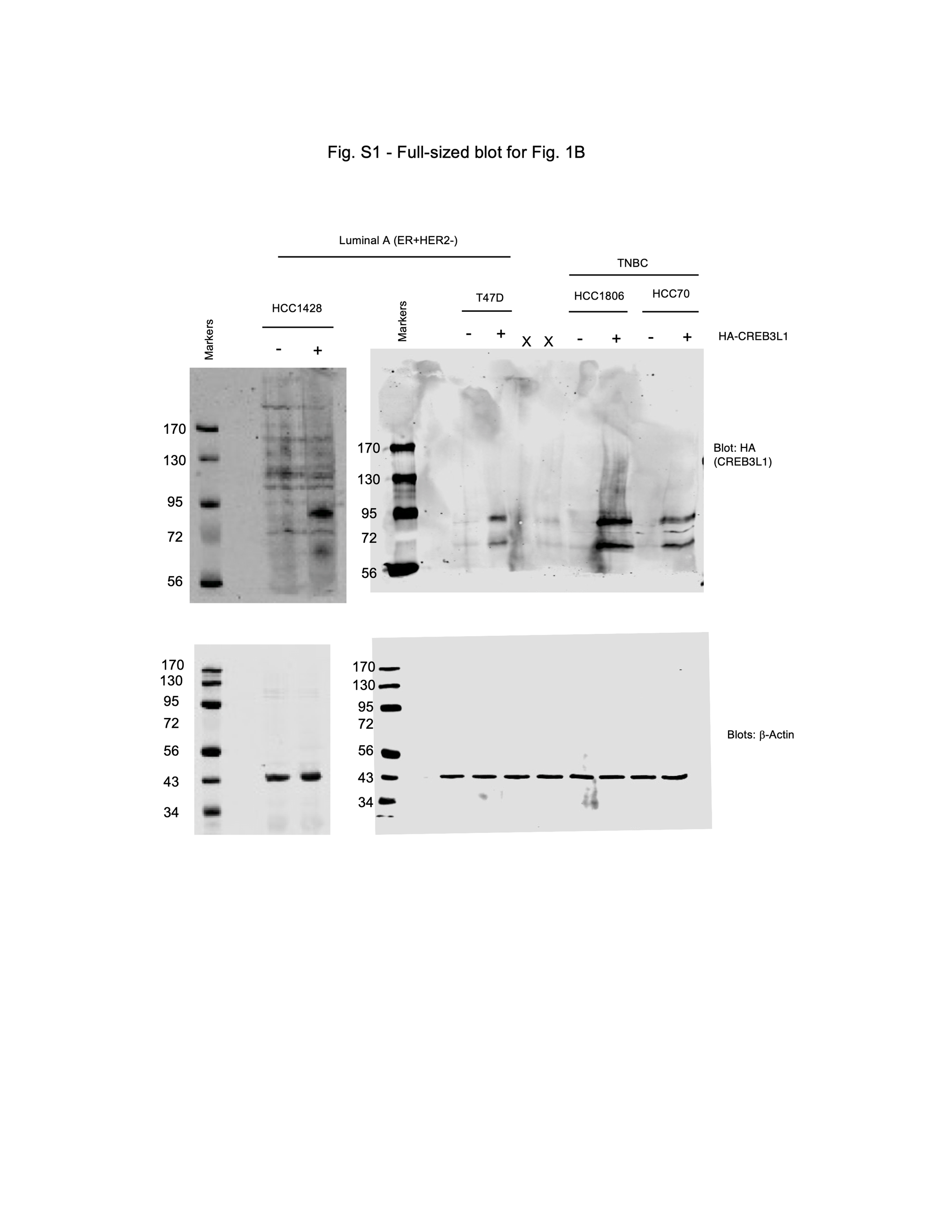

Supplement: S1 Fig — (TIF) [file pone.0271090.s001.tif]

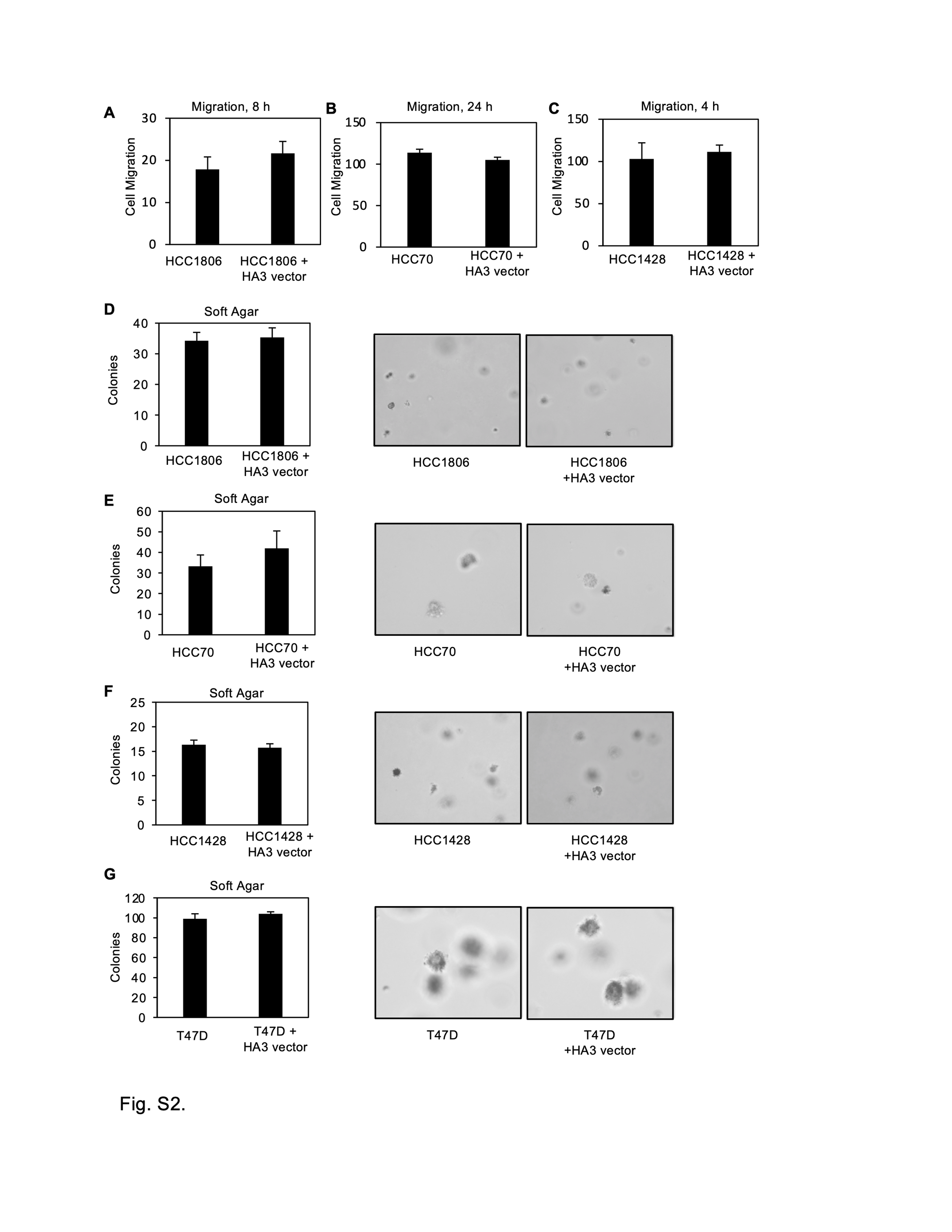

Supplement: S2 Fig — (A-C) The indicated cells were allowed to migrate towards 10% FBS through Boyden chambers for the indicated times and were fixed, stained and counted. Mean ±SEM from at least 2 independent experiments, each with duplicate determinations. (D-G) Growth in soft agar was determined after 21 days. Mean ±SEM from at least 2 independent experiments, each with duplicate determinations. In each case, there were no significant differences between the parental and vector control cells. (TIF) [file pone.0271090.s002.tif]
